# Will Mary Jane Leave You Heartbroken? Marijuana Use and Risk of Takotsubo Cardiomyopathy

**DOI:** 10.7759/cureus.5987

**Published:** 2019-10-24

**Authors:** Alexandra M Sanchez, Susie Sennhauser, Matthew R Lozier, Joshua M Purow

**Affiliations:** 1 Internal Medicine, Holy Cross Hospital/ University of Miami, Fort Lauderdale, USA; 2 Cardiology, Mayo Clinic Jacksonville, Jacksonville, USA; 3 Cardiology, Jim Moran Heart and Vascular Research Institute at Holy Cross Hospital, Fort Lauderdale, USA

**Keywords:** takotsubo, cardiomyopathy, marijuana, catecholamines

## Abstract

Takotsubo cardiomyopathy (TTC) is prevalent in 2% of patients who present with symptoms suggestive of acute myocardial infarction. It may be triggered by stressful events, resulting in catecholamine surges, myocardial stunning, and disturbances in contractility. TTC in males has been associated with marijuana use and leads to a fivefold increased risk of cardiac events. Marijuana is thought to generate a similar surge in catecholamines leading to tachycardia and elevation of both systolic and diastolic blood pressure. The question remains whether this catecholamine surge is sufficient enough to cause TTC. It is apparent a correlation between marijuana use and TTC may exist. Exogenous cannabinoid stimulation may lead to myocardial stunning via stimulation seen with hypercatecholaminergic states. Understanding the risk factors involved and increasing awareness of cardiovascular complications related to cannabinoid substances becomes more relevant as its use is increasing both recreationally and medically.

We present a case of a 50 year-old African-American male with hypertension and regular marijuana use who presented with chest pain radiating to the back. Due to abnormal electrocardiogram and positive cardiac biomarkers concerning for acute coronary syndrome, the patient underwent subsequent coronary angiography that showed no significant coronary obstruction; however, left ventriculogram showed the characteristic apical ballooning of TTC. Our case highlights the pathophysiological mechanism suspected to trigger TTC.

## Introduction

Takotsubo cardiomyopathy (TTC), also known as left ventricular apical ballooning syndrome or stress induced cardiomyopathy, is characterized by acute reversible left ventricular dysfunction in the absence of significant coronary artery disease (CAD) [1]. This cardiac condition, which may be triggered by stressful events, results in catecholamine surges and subsequent myocardial stunning presenting as transient disturbances in contractility [2-3]. Marijuana use generates a chemically similar surge in catecholamines leading to tachycardia and elevation of both systolic and diastolic blood pressure; however, it has yet to be determined whether this catecholamine surge is sufficient to cause TTC [1-3].

## Case presentation

**Figure 1:**
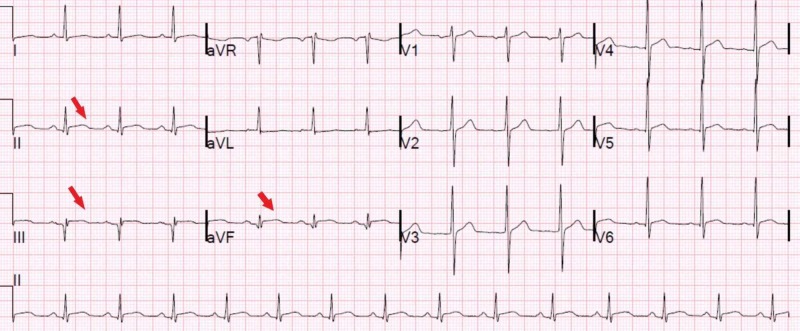
Initial electrocardiogram showing inferior ST elevations (red arrows), with troponins elevated at 1.270ng/dL

**Figure 2:**
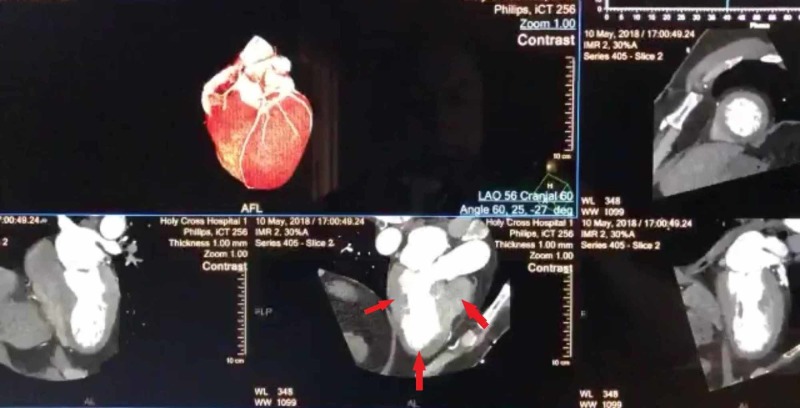
Coronary Computed Tomography Angiography with decreased perfusion in left ventricular apical segment and dyskinesia The red arrows show a still image of a coronary computed tomography angiography demonstrating decreased perfusion to the left ventricular apical segment with an estimated ejection fraction of 28%.

**Figure 3:**
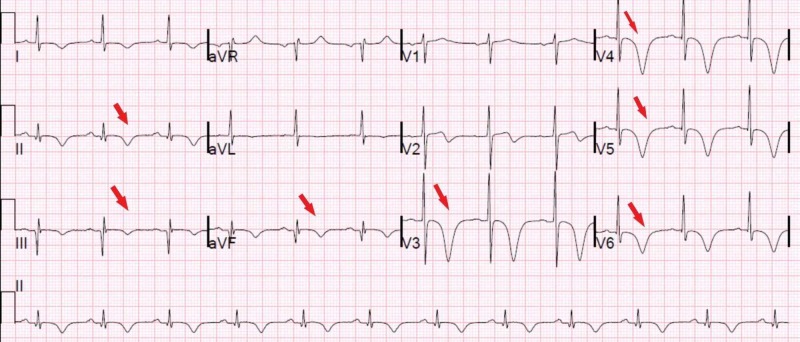
Electrocardiogram showing previous inferior infarct; anteroseptal and lateral ST-T wave abnormalities status post imaging with troponins peaked at 31.3 ng/dL The red arrows reflect post-ischemic T-wave inversions.

A 50-year-old African-American male with regular marijuana use and a history of hypertension and hyperlipidemia presented with acute onset, retrosternal chest pain radiating to the back. Blood pressure upon admission was markedly elevated at 217/136 millimeters of Mercury (mmHg) with new dynamic electrocardiogram (ECG) abnormalities noted in the inferior leads [Figure 1]. Serial troponin levels were obtained rising and peaking at 31.3 nanograms per deciliter (ng/dL) (normal range: 0-0.045) with positive creatinine kinase-myoglobulin (CK-MB) index. Emergent cardiac computer tomographic angiographic (CCTA) imaging was obtained. Images revealed mild non-obstructive CAD as well as a left ventricular ejection fraction (LVEF) of approximately 28% with global left ventricular hypokinesis and apical dyskinesis. No aortic dissection was noted. A presumptive diagnosis of TTC was made based on these CCTA findings [Figure 2]. Due to abnormal ECG [Figure 3] and positive cardiac biomarkers concerning for acute coronary syndrome (ACS), the patient underwent subsequent coronary angiography that showed no significant coronary obstruction [Videos 1-2]; however, left ventriculogram showed the characteristic apical ballooning of TTC [Video 3]. 

**Video 1:** Coronary catheterization demonstrating no LAD obstructive disease LAD: Left anterior descending artery The video above shows diffuse mild and non-critical disease to the LAD.

**Video 2:** Coronary catheterization demonstrating no RCA obstructive disease RCA: right coronary artery The video above shows diffuse mild and non-critical disease to the RCA.

**Video 3:** Left ventriculogram showing characteristic apical ballooning in takotsubo cardiomopathy

This case was presented as an abstract at the American College of Cardiology 68th Annual Scientific Session and Exposition Conference in March 2019 (https://www.sciencedirect.com/science/article/pii/S0735109719333170?via%3Dihub). 

## Discussion

TTC accounts for approximately 2% of patients initially suspected of an ACS with roughly 90% occurring in women [1]. Some studies have found that women with TTC were noted to have an acute stressful event, while TTC in men was more commonly linked with active marijuana use [Abstract: Singh A, Agrawal S, Fegley M, Manda Y, Nanda S, Shirani J. Marijuana (Cannabis) Use is an Independent Predictor of Stress Cardiomyopathy in Younger Men. American Heart Association Scientific Sessions; November 2016]. The diagnosis should be considered in the differential of patients with dyspnea or chest pain in the setting of active marijuana use with symptoms mimicking acute myocardial ischemia (MI) [Abstract: Singh, November 2016]. Typically, coronary angiography shows no significant obstructive CAD; however, it is important to note that the risk of an acute MI increases nearly fivefold within an hour of cannabis exposure [2-4]. A definite link between marijuana use and a non-ischemic cardiomyopathy is difficult. As in this case, patients often have multiple factors that may contribute to the event, but marijuana use must be considered. Consequently, in such cases, a comprehensive history must be obtained including substance use.

The endocannabinoid system (ECS), composed of the cannabinoid receptors types 1 and 2 (CB1 and CB2) for marijuana's psychoactive ingredient Δ9 tetrahydrocannabinol (Δ9-THC), is an important mediator between the hypothalamic-pituitary-adrenocortical (HPA) axis and stressful conditions [5-6]. Stress is recognized as an adaptive response to stressful stimuli, which activates the HPA axis and the sympathetic adrenergic system. It has been observed that the apex contains the highest β-adrenergic receptors (βARs) and lowest sympathetic nerve density [5]. The presence of ventricular βAR gradient results in increased apical responsiveness to catecholamines, mainly epinephrine, which at high levels can have negative inotropic impact through the β2AR6. These observations support the hypothesis that regional differences in β2AR could explain the myocardial response to increased catecholamine circulation seen in TTC. Similarly, cannabinoid receptors (CB-1 receptors) are present in human cardiac muscle, allowing for cannabinoids to impact myocardial function [7]. It has been shown that endocannabinoids and THC elicit CB-1 receptor-mediated bradycardia, hypotension, and decreased cardiac contractility [8-9]. While the exact mechanism is still not fully understood; it is hypothesized that ‘classical’ and endothelial cannabinoid receptors prompt the release of nitric oxide resulting in a vasodilatory effect as well as inhibition of the peripheral nervous system and activation of the sympathetic nervous system [10-11].

Takotsubo cardiomyopathy has often been associated with hypercatecholaminergic states. A recent study showed that patients with TTC had catecholamine levels that were 2 to 3 times greater than patients with classic MI's, resulting in microvascular spasm and myocardial stunning likely secondary to beta-receptor mediated sympathetic stimuli [3,12-14]. Regarding the role of marijuana in TTC, it has been theorized that the endocannabinoid system may also cause physiologic stress as illustrated above, leading to cardiomyopathy.

Given the similarity in the pathophysiology of TTC and the mechanism of action of cannabis, it is important to further investigate the potential relationship between marijuana use and stress cardiomyopathy. This becomes even more relevant as the use of cannabis and cannabinoid substances increase both recreationally and pharmaceutically. As seen with endocannabinoid stimulation, a similar mechanism could apply to exogenous cannabinoids such as marijuana. Acute use is associated with tachycardia, while chronic use is linked with hypotension and bradycardia [15-16].

## Conclusions

A true pathophysiologic explanation for TTC remains unclear; however it is apparent that it is not a homogeneous process. Through mechanisms discussed above, a strong correlation between marijuana use and TTC is suspected. This may indicate that exogenous cannabinoid stimulation may lead to myocardial stunning via more intense stimulation. Due to the fact that recreational and medical use of cannabis has sharply increased in the recent years, further research is warranted. It is necessary to increase awareness regarding the increased risk of cardiovascular complications associated with cannabis use.
